# Notes and News

**Published:** 1902-07-12

**Authors:** 


### NOTES AND NEWS.

The peace-making dinner and reunion of the obstetricians and gynæcologists of the Empire, under the presidency of Sir John Williams, took place at the Hôtel Métropole on June 24th.

It is proposed that the British Medical Association shall meet in 1903 at Swansea. Dr. T. D. Griffiths has been recommended for President-Elect. The last time the Association met in Swansea was in 1853.

In the report of the Army Medical Department for 1900, which has just been issued as a Blue-book, a curious instance of the transport of infection for a long distance is recorded. It is stated that the health of the troops in Egypt was good on the whole, but that an outbreak of scarlet fever occurred among the 3rd batt. Seaforth Highlanders, at Cairo, during the latter portion of the year. This outbreak was attributed to parcels sent from localities in Scotland where the disease was rife. The battalion was placed under canvas, and all parcels were disinfected, with satisfactory results.

To carry out the instructions of the new warrant in regard to the instruction and examination of officers of the Royal Army Medical Corps, a scheme for a temporary medical staff college in London is now in preparation. It will involve the provision of two sets of courses, one for officers entering the service, the other for officers who are approaching the time for examination for promotion. The courses for those entering will for the present be held in laboratories to be rented from the Royal Colleges on the Thames Embankment, but those for officers on promotion will have to be arranged for elsewhere. In the future it is probable that a military hospital will be erected in London in connection with the permanent Staff College at which instruction in subjects specially connected with military medicine will be given, while arrangements will be made with the civil hospitals for clinical work and special courses on other subjects. In any case officers granted study leave will not be turned loose to spend their time at their own discretion.

The vacancy in the coronership for the South-Western Metropolitan District occasioned by the death of Mr. Braxton Hicks is to be filled by the appointment of Mr. Troutbeck who is also coroner for Westminster.

Those who, while fond of congresses do not consider August the most appropriate month for such gatherings, will probably find the Congress of Medicine, which is to be held in Cairo next December, very attractive. An English committee under the presidency of Sir Frederick Treves has been formed to forward the interests of the Congress, and Sir Thomas Smith, Dr. Stephen Mackenzie, Sir Laudon Brunton, Dr. Savage, Sir Victor Horsley, Dr. James Taylor, and Mr. Watson Cheyne have consented to act upon it. Dr. W. Page May is honorary secretary of this committee.

The Imperial Vaccination League, an organisation which was formally constituted on the last day of last month, aims at giving a general support to movements in favour of vaccination by means of leaflets, lectures, meetings, and the like. Its president is the Duke of Fife. The treasurer is Lord Avebury, and the provisional committee consists of Dr. Thresh, Dr. J. G. Glover, Dr. J. D. Acland, Dr. Dudfield, Mr. R. Brudenell Carter, Dr. E. Garrett Anderson, the Rev. H. L. Paget, Mr. John Carrington, and Mr. Greenwood. The League intends at once to collect opinions as to what amendments of the Vaccination Act should be urged upon the Government when the time comes for dealing with the subject.

At an Extraordinary General Meeting of the British Medical Association, held at Exeter Hall on July 9th, new regulations for the governance of the Association were finally accepted. Now, at last, the executive (and with the executive we may perhaps include the editor of the *Journal*) will be able to ascertain directly, and by constitutional means, the mind and the wish of the members. Hitherto the fashion has been to blame the Council for everything, but henceforth whatever may be done in the name of the Association will be the direct outcome of the action or the non-action of its members, and they will have to bear the blame. The machinery, however, is cumbrous enough to dishearten most reformers.

The ballot for the election of four members of the Council of the Royal College of Surgeons of England resulted as follows:—

| | Votes. |
|---|---|
| Mr. Howard Marsh ... ... ... | 415 |
| Mr. John Hammond Morgan ... ... | 393 |
| Mr. Henry Hugh Clutton ... ... ... | 311 |
| Mr. Charles William Mansell Moullin ... | 293 |

The numbers for the unsuccessful candidates were as follow:—

| | |
|---|---|
| Mr. Jordan Lloyd ... ... ... ... | 241 |
| Mr. John Bland-Sutton ... ... ... | 228 |
| Sir William Henry Bennett ... ... | 205 |
| Mr. Clinton Thomas Dent ... ... ... | 85 |

Mr. Marsh was declared duly re-elected and Messrs. Morgan, Clutton, and Moullin duly elected.

